# Predictors of COVID-19 Vaccination Campaign Success: Lessons Learnt from the Pandemic So Far. A Case Study from Poland

**DOI:** 10.3390/vaccines9101153

**Published:** 2021-10-09

**Authors:** Marcin Piotr Walkowiak, Dariusz Walkowiak

**Affiliations:** 1 Department of Preventive Medicine, Poznan University of Medical Sciences, 60-179 Poznań, Poland; MarcinWalkowiak@wir.pl; 2 Department of Organization and Management in Health Care, Poznan University of Medical Sciences, 60-356 Poznań, Poland

**Keywords:** COVID-19, vaccination, trust in vaccine, public health, health policy

## Abstract

The high effectiveness of a vaccination-promotion campaign, which may be measured by the number of those successfully convinced to get vaccinated, is a key factor in combating the COVID-19 pandemic. This, however, appears to be linked to the precise identification of the underlying causes for vaccine hesitancy behaviours. Based on a regression model (adjusted R^2^ of 0.78) analysing 378 sub-regions of Poland, we showed that such behaviours, even when going against the party agenda, can be indirectly yet precisely gauged predominantly through voting patterns. Additionally, education and population density were found to be positively related to low vaccine hesitancy, while markers of social exclusion, both external (employment rate) and psychological (voter turnout) ones, affected it negatively. In the second, follow-up part of our study, which analyses the changes that took place in two months (adjusted R^2^ of 0.53), we found a further increase in vaccination rate to be positively related to the number of those already vaccinated and to the political views of the population, and negatively related to its level of education. In both cases, there was a surprisingly weak relationship between the potential markers of accessibility and vaccination rate. In spite of the known overall differences in vaccination rates for different age and sex groups, these variables did not have any additional informative value in explaining the observed regional differences.

## 1. Introduction

There have been a lot of surveys exploring the coronavirus disease 2019 (COVID-19) vaccine acceptance and its determinants since March 2020. Some of them were carried out at a time when a vaccine was a distant solution, and others during the vaccination campaigns in individual countries. They were conducted in Poland [1,2], the United States [3,4,5,6,7], Ireland and the United Kingdom [8,9], Australia [10], Canada [11], France [12], Italy [13,14,15], Portugal [16], Finland [17], Greece [18], China [19,20] and Japan [21]. As a result of multicentre cooperation, surveys in seven European countries [22], seven European countries and the United States [23], three Asian and five African countries [24], 23 Arab countries and territories [25] and 19 countries with a high COVID-19 burden [26] have been conducted. Some scholars searched for regularities which may have influenced people’s behaviour for particular countries studied, while others looked for more general ones. The results obtained in multicentre studies indicate serious differences between countries [8,23,26,27,28]. A wide range of results have been presented. The results of some studies were inconsistent with those of others, which of course is understandable given the different methodologies or research groups, as well as the issues studied and research questions asked. Several of the above-mentioned studies indicated that males [2,10,12,20,21,24], older individuals [13,14,16,18], those with a higher income and/or education level [11,12,14,16,24,29] or those having been vaccinated against the flu previously [13,18,20,25,27] were more likely to accept a COVID-19 vaccine. The influence of many other factors, such as higher trust in government decisions [10,28], faith in science [15,28], political beliefs [4,5,7], perceived effectiveness of a COVID-19 vaccine [4,20], personally knowing someone who was infected by COVID-19 [12], living in a region with higher rates of COVID-19 infections [25], and living in smaller communities [13] were associated with a higher willingness to be vaccinated. Factors associated with lower willingness to be vaccinated included conspiracy beliefs [2,8,15], being unemployed [6], having suffered financial consequences during COVID-19 pandemic [11] or living in disadvantaged areas [10]. The existence of racial or ethnic differences in individual countries was also shown [4,6]. Variations in the level of acceptance of vaccinations have also been observed over time [17].

The lack of willingness to accept a COVID-19 vaccine in Poland is a cause for concern. A survey conducted just before the outbreak of the pandemic in 18 European countries [30] showed that Poland was in seventeenth place as far as vaccine confidence among parents is concerned. A global survey of 19 countries, published in Nature Medicine, found Poland to be, again, second-to-last in its ranking of the percentage of those willing to take a COVID-19 vaccine if proved effective [31]. De Figueiredo et al. [32], in a large-scale retrospective temporal modelling study published in The Lancet, found in Poland a decrease in the confidence in vaccination between 2018 and late 2019. However, in their study on the psychological roots of anti-vaccination attitudes in 24 countries, Hornsey et al. [33] found that the negative attitudes toward vaccination in Poland, compared to other countries, are not as easily attributable to anti-vaccination ideology. Thus, the challenge for a scholar researching vaccination attitudes in Poland is how else to account for the observed phenomenon, what determines the unsatisfactory level of vaccination against COVID-19, and what else can still be done to bring the vaccination levels of the population to the levels that are safe from the point of view of virus transmission. Vaccinations, unfortunately, do not follow the assumptions of scientists and will probably not ensure population immunity while the current trends are maintained.

Our present study markedly differs in its method from the majority of sociological research discussed above, which used surveys of selected samples of the population. Instead of sampling and then asking respondents questions, we decided to work on big numerical data and to identify differences in certain sociodemographic parameters of particular population subgroups in the hope of finding correlations between subgroup characteristics and the actual responsiveness to vaccination campaigns. Moreover, and in contrast to sociological surveys, which were feasible (and actually done) even before the outbreak of the pandemic, our type of research can only be done at this moment, when the effectiveness of national vaccination campaigns can be quantified and assessed.

## 2. Methods

### 2.1. Study Design

Data on the number of vaccinated inhabitants of Poland, published online by the Polish Ministry of Health [34], were collected for the following days: 30 June and 31 August 2021. The Ministry database provided a snapshot of the daily and aggregated number of first and second shot vaccinations, calculated separately for each county (“gmina”), with the data aggregated, for study purposes, to sub-regions (“powiat”), which is an administrative unit formerly classified in the European Union (EU) nomenclature as LAU-1 and NUTS-4. Poland is divided into 380 such heterogeneous sub-regions, which can include a city, a suburb surrounding a city, or an area of countryside. This study analysed, based on regression models, which local factors explain differences in vaccination rate. Data collection is ongoing, though the number of daily vaccinations has already peaked on 3 June 2021, with an increasing number of empty slots and slowly waning interest among remaining potential patients, and since 7 July onward, vaccination of 12-year-olds has begun.

### 2.2. Outcome

The measured outcome is the share of population in each sub-region that until 30 June 2021 received at least one dose of any COVID-19 vaccine, and subsequently the percentage of the population that received their first shot between 30 June and 31 August 2021.

### 2.3. Independent Variables

Based on prior studies on the willingness to get vaccinated, demographic and geographic data were taken from the Polish Statistical Office database [35], including gender imbalance and age (measured as the numbers of people above 60 and 70 years who in Poland received priority access to vaccine) as of the end of 2020. To test the possibility of a direct or indirect impact of social exclusion, the number of registered unemployed for longer than half a year in the age group 18–24, or longer than a year among the people aged 55–64, as of 31 December 2019, i.e., before the pandemic, was used as a proxy indicator.

Prior local severity of the pandemic, which could have affected the perceived threat level, was gauged using the number of local COVID-19 deaths according to the Ministry of Health database [36]. Because of the lack of sufficiently precise geographic location for the data logged in 2020, as well as the lag in data recording, the data on the deaths from 31 December 2020 to 3 July 2021 were used. As a proxy indicator, we used both the general number of deaths attributed to COVID-19 and the number of cases where it was the only stated cause of death, as those could have had a higher psychological impact.

Some geographically detailed data had to be taken from the 2011 census, which—because of the changes in the administrative division—led to the elimination of two sub-regions from the model. This census was the source of the information on the share of population with a higher education level, on the employment ratio, and on the percentage of households without access to the sewage system, as a proxy indicator of the number and geographic localisation of the people who live in underserved or remote areas.

The study also analysed potential external obstacles, unrelated to subjective perception. The number of stationary vaccination sites for each sub-region of Poland was obtained from the Ministry of Health website [37]. Additionally, the Ministry also collected the data on the number of vaccinated people by sub-region of vaccination site [38], which we used to identify the sub-regions which in the early phase of vaccination rollout had some deficit or surplus, which was otherwise hard to pinpoint.

Based on prior studies on anti-vaccine attitudes, ideological views were better determinants than demographic factors [33]—scepticism towards vaccines was correlated with other with conspiratorial beliefs, contrarianism and a combination of individualism and a hierarchical worldview. Additionally, studies of Polish YouTube channels showed a linkage between right-wing ideas and vaccine scepticism [39]. Under the Polish voting system, the first round of the presidential elections allows voters to reveal their true ideology without much potential for strategic voting, while the second round compels them to select one of the two main political forces. As one variable, support of the right-wing populist [40] Law and Justice party candidate in the second round of the 2020 presidential elections was selected [41]. He retained the popular mandate, while his party’s response was a rather standard one, with lockdowns, mask mandate and, ultimately, vaccine rollout. As a second proxy indicator, support in the first round for the candidate of the Confederation of Liberty and Independence, a coalition of strongly right-wing parties, was used as a political marker of a potentially less distrustful and more individualistic type of scepticism. The position of the Confederation could be better expressed as scepticism not towards vaccines per se but towards the concept of public health [42], especially when it infringes on individual freedoms. It was the only political force that voted against the law allowing the lockdown [43], encouraging its voters to make an informed decision after consulting a medical professional they trust [44], and vehemently objected to the vaccination of children based on individual cost–benefit grounds [45]. The voter turnout in the second round of presidential elections was used independently as a general marker of sociopolitical engagement. Additionally, as a political marker of adherence to the ideas of the hypothesized anti-vaccine movement, we also used the number of votes in the first round of the 2015 presidential election for Paweł Kukiz, the leader of the protest party Kukiz 2015, which is generally classified as populist right-wing [40], though this term oversimplifies its diverse electorate, as the majority of the voters were unable to place themselves on a right–left axis and only 22% saw themselves as right wingers [46]. Kukiz brought into parliament a few anti-vaccine activists, although subsequently they were even challenged by their own fellow party members [47]. In the case of both the Confederation and Kukiz 2015, it rather looked as if part of the electorate of both parties had already been sceptical, while the parties were trying to accommodate as wide a spectrum of supporters as possible.

### 2.4. Statistical Analysis

A simple linear regression model was selected and subsequently validated in a spatial lag regression model, where the average result from the 6 nearest sub-regions was used as a proxy for some regional factors. Whenever possible, relative values were used to adjust for size differences among sub-regions. While it was possible to use the logarithms of some variables, it would have led to lower predictive value, and there were no grounds to assume multiplicative relation. The variables were often intended to measure similar phenomena (such as political views or social deprivation), and variance inflation factors (VIF) were calculated to eliminate the risk of collinearity or low predictive value when applied in conjunction with others. As the situation after two months was relatively similar, only the factors explaining the increase were analysed.

The data obtained from official sources were verified and checked for completeness, quality and consistency. Then, they were coded and exported into a statistical package for model creation in GRETL 2019d and for subsequent testing for spatial relations in GeoDa 1.18.0. Maps were generated using MapChart (mapchart.net). A 5% level of significance was used for all tested hypotheses.

## 3. Results

### 3.1. Vaccination Rate for 30 June 2021

**Figure 1:**
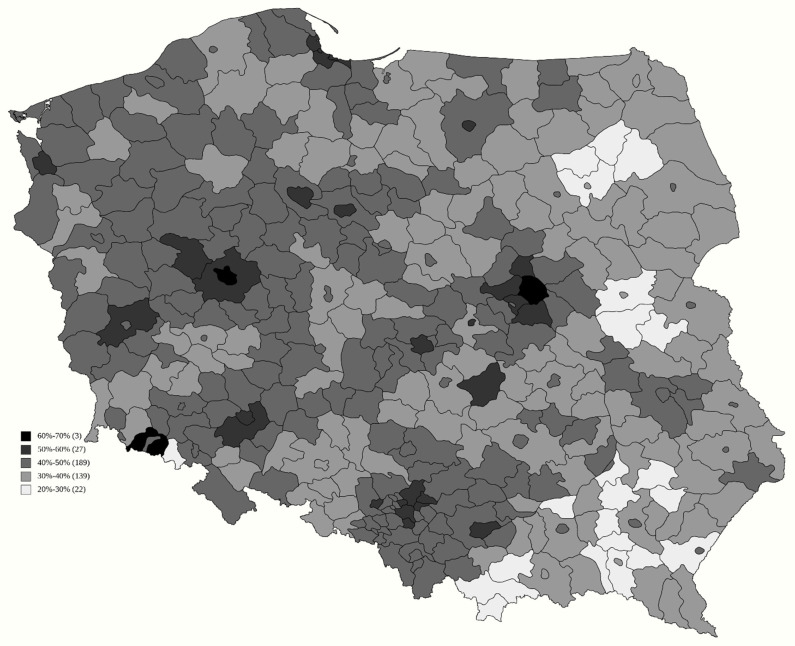
Percentage of Polish inhabitants receiving their first COVID-19 vaccine dose until 30 June 2021.

The generated model (Table 1) had very high predictive value of adjusted R^2^ of 0.78, and all but two variables had p-value orders of magnitude below even 0.001 for explaining the vaccination rate, which is presented in Figure 1. Each single percentage increase in support for the Law and Justice candidate in the second round reduced the number of vaccinated by 0.201 percentage points (p.p.). Nevertheless, this value should be seen in perspective, as voter turnout in the second round increased the vaccination rate by 0.308 p.p. Even more pronounced was the result of support for the Confederation in the first round, as each single percentage increase in their support reduced the vaccination rate by 1.678 p.p. Surprisingly, support for Kukiz in the 2015 election was not a predictive factor.

The percentage of people with a higher education level also turned out to have high predictive value, as each single percentage increase in the number of such people increased the number of those vaccinated by 0.416 p.p. A similar, though weaker (0.133 p.p.) impact was found for the employment ratio. In spite of the political factors and education levels, already favouring cities, on top of that, a higher population density also had a positive influence on the number of the people vaccinated. Factors such as the number of vaccination sites in relation to population or region area, or even the percentage of people who had to travel to another sub-region to vaccinate, turned out not to generate additional informative value.

The local severity of the pandemic, measured by the number of COVID-19 deaths per 1000 inhabitants, lacked any predictive value, while there was a minute impact of the number of deaths where COVID-19 was, according to the death certificate, the sole reason for death. Each such case per 1000 increased the number of those vaccinated by 0.015 p.p. Local differences in age or sex ratio did not have any additional predictive power.

### 3.2. Vaccination Rate Increase between 30 June and 31 August 2021

**Figure 2:**
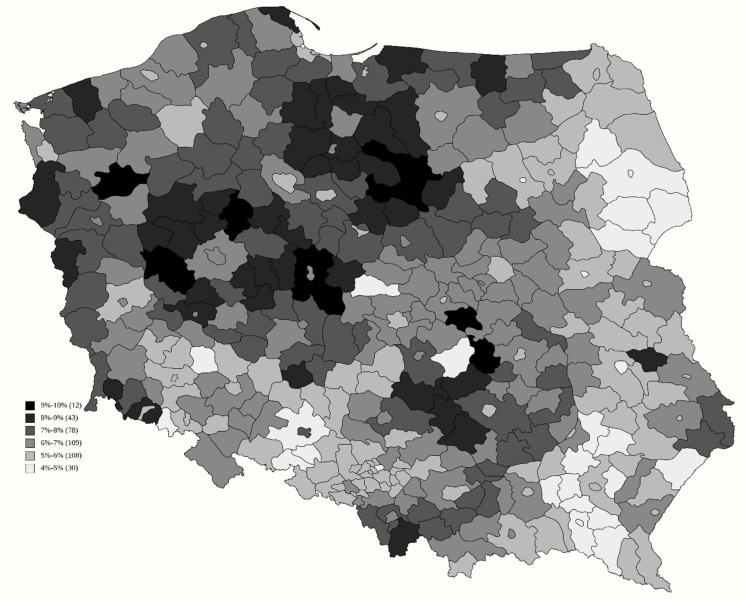
Percentage of Polish inhabitants receiving their first COVID-19 vaccine dose between 30 June 2021 and 31 August 2021.

Two models were created to find factors explaining the further vaccination rate increase from 44.2% by an additional 6.6 p.p., as presented in Figure 2, based on whether the prior vaccination rate can be used as an explanatory variable, with an adjusted coefficient of determination R^2^ of 0.53 (Table 2) and 0.49 (Table 3). The impact of this variable was 0.060 p.p. The indirectly gauged ideology was still a highly relevant factor, as support for the Confederation had a negative impact of −0.300 p.p. and −0.463 p.p., while support for Kukiz—by −0.120 p.p. and −0.105 p.p., respectively. The share of people above 70 years of age also reduced inoculation rate by −0.173 and −0.206, while the impact of a higher education level actually reversed the sign to −0.107 p.p. and −0.047 p.p., respectively. Additionally, in both variants of the model, a higher number of inhabitants per vaccination site actually increased the chance of getting vaccinated.

### 3.3. Spatial Lag Analysis Models

The models were tested for spatial lag, which could affect the assumption of independence of the observations. The spatial lag variable of the average vaccination rate was added to the model from Table 1, with the result presented in Table 4.

In spite of the spatial lag variable having a high coefficient of 0.528, all the variables used maintained their sign. However, two variables—population density and employment to population ratio—lost their statistical significance. In comparison with the original model, the impact of these two variables actually increased: deaths per 1000 where COVID-19 was the sole reason for death reached an impact of 0.019, while the percentage of people with a higher education level reached an impact of 0.639. All the political variables had their predictive value seriously reduced—voter turnout reduced to 0.126, vote for Law and Justice reduced to −0.101, and vote for Confederation reduced to −0.951.

An analogous spatial lag test was applied to the model from Table 3, and is presented in Table 5. In this model, there was also a serious impact of the spatial lag variable, as its coefficient was 0.595. Nevertheless, all the variables used maintained their sign. The number of people per vaccination site clearly lost its statistical significance, as did the prior vaccination rate, but with the latter variable, it was a matter of an arbitrary threshold point, because in that case, its *p*-value was 0.066. The absolute values of coefficients of all the remaining variables decreased: vote for Confederation reduced to 0.174, vote for Kukiz reduced to −0.057, the share of people with a higher education level reduced to −0.070 and the share of those over 70 years of age reduced to −0.114.

## 4. Discussion

Our findings are consistent with earlier survey-based Polish studies [2], showing that there is a clear relationship between vaccination rate and the following: conspiratorial views, religiosity, social deprivation and education. However, there was one serious divergence, as in our model, demographic data such as age or sex were not relevant on their own, most probably being overshadowed by the political views correlated with them. However, the results are different from those obtained by Furman et al. [48], who found a high and rather uniform level of trust in the safety of mandatory vaccines over effectively all tested demographics. Such serious difference most likely shows that the observed COVID-19 vaccine hesitancy is actually only weakly related to earlier (pre-pandemic) anti-vaccine movements.

The relationship between vaccine hesitancy and support for a right-wing ideology should be seen in context. If we divide Polish society, according to the stance during the second round of the 2020 presidential election, into three groups of comparable size, the most vaccinated was the electorate of the Civic Platform (EU faction: the European People’s Party, which by EU standards is moderate right), while the electorate of the Law and Justice (EU faction: the European Conservatives and Reformists) was actually the middle group, and the least vaccinated group were those who did not participate in the elections. The same order would have appeared had we arranged these groups on the basis of their trust level, understood as the willingness to cooperate and the disbelief that some hidden forces were actually in charge [49], while prior studies pointed to a very strong relationship between trust and the willingness to follow epidemiological restrictions [28,50].

This may partially explain why subsequent pro-vaccine campaigns of Law and Justice politicians were of very limited success—they had to primarily convince people who actually did not even trust them enough to vote for them. While it was possible to show the relationship between vaccination rate and support for the parties that try not to alienate the sub-segment of their electorate that is vaccine-sceptical, this was clearly only part of the picture. This small but highly active group may overshadow the bigger but passive group that, according to our model, were those lacking faith in the state institutions so much that they even did not vote for right-wing populists, and their lack of faith was reinforced by their prior experience of social exclusion, as they had been left behind because of their lack of education and their coming from economically destitute regions with no job opportunities.

The lack of impact of the local number of COVID-19 deaths can either mean that these deaths were ignored, or that their impact was cancelled out by those who acquired immunity through infection this way and felt no need to inoculate. Nevertheless, there was a minor impact in those cases where COVID-19 was the sole reason of death, suggesting that that fact may had convinced those who personally knew the deceased.

The existing body of research on vaccine hesitancy has shown in Poland a high and relatively stable level of scepticism that even preceded the actual vaccine rollouts [1,28,48,51]. The models implied the existence of even more long-term factors—demographic variables from 2011 or voting patterns from 2015 still had high predictive value in 2021, suggesting underlying patterns unrelated to any recent public opinion swings. Their existence was further confirmed through the analysis of spatial lag, which in the model explaining prior vaccination rate reduced the significance of all variables except for the share of people with a higher education level and the number of deaths caused solely by COVID-19. This implies that these variables were clearly related to local, sub-regional factors, while the remaining variables of a sociopolitical and geographical nature were, to a large extent, facets of some regional phenomena.

Variables related to the access to a vaccination site, including population density (which may partially reflect physical proximity) or the number of inhabitants per vaccination site, had at most a weak impact, and clearly lost statistical significance when taking into account the spatial lag. This implies that even though in the early phase the phenomenon of travelling to different regions for inoculation was clearly visible, in the long run, the key barriers were not physical but psychological in nature.

The follow-up analysis brought some deeper insights. The expected catching-up of the sub-regions that were not successful in the beginning did not happen. There clearly existed a spatial lag in these phenomena, and presumably a time lag as well. This seems to indicate that whichever regional factors had been relevant to begin with retained their importance. However, as other explanatory variables dramatically changed, it could also be interpreted that, in accordance with social proof theory, in an ambiguous situation, people tended to base their decision on the decisions made by those prevalent in their surroundings [52,53], and social networks should span beyond sub-region borders. This would explain why out of the political variables, only the support for fringe parties remained relevant, as their electorate would be less likely to bow to social pressure. Age over 70 became a predictive factor, though it mostly meant that this demographic was already mostly vaccinated. The impact of education reversed the sign, suggesting that this group had been early adopters and currently were mostly vaccinated.

Since, based on this study, the key target group to convince are not the opponents of vaccines but the people generally distrustful of the establishment, there is a clear need to shape policies accordingly. According to the EU member states’ vaccination rates data [54], the most obvious division line runs along the former Iron Curtain, with post-Communist countries clearly underperforming. Even if, asked about their confidence in particular vaccines, Polish respondents expressed mixed feelings about AstraZeneca (which at that time had a very bad press), they still did not rate it any worse than barely known vaccines from Asia. By contrast, they expressed complete distrust towards Russian Sputnik V [51], which might be consistent with long-standing distrust of anything associated with (post-)Communist Russia. Judging from the lingering lack of trust in former Communist countries, which affects vaccination rates, one should be wary of well-intentioned policies that may be understood as censorship or authoritarianism by the target audience. Heavy-handed policies combating “misinformation” and “conspiracy theories” can be of limited use when official messages are subject to 180-degree reversal, as for example in the initial government stance, discouraging the general population from wearing masks [55,56,57,58,59]. There exists a wide body of literature on how a company should regain trust after bad publicity—Xie and Peng [60] demonstrated that customers not only expect policy correction and information, but actually the key psychologically important part is apologising for past misdeeds. Similarly, strategies designed to rebuild trust are more effective than attempts to deny or diminish impact [61,62]. Judging from growing civic disobedience in countries with more aggressive policies, and from the signs that further cooperation may be required for additional inoculation against emerging virus variants, one should seriously think whether well-intentioned policies would not be misinterpreted and counterproductive among the least trustful segment of society.

Additionally, when aiming their campaigns at the most distrustful, the government should rely more on those considered trustworthy, as politicians and the media are clearly deemed to have low credibility in Poland [63]. Since charity organisations are on top of the Polish trust ranking, they should clearly be involved more. Moreover, because those who do not trust official institutions still put faith in their close circle of family and friends, higher importance should be given to indirect campaigns meant to convince a vulnerable vaccine-hesitant relative, especially when the model already implies the existence of some kind of social pressure.

## 5. Conclusions

The created models confirm the relationship between political views and vaccine hesitancy in a method that unlike prior studies is not vulnerable to desirability bias and other survey biases. What is more, this methodology allowed us to analyse how views translate into actual behaviour with the possibility of not being motivated enough to overcome obstacles, or remaining personally sceptical, though yielding to social pressure. It also enabled us to show that on top of the already noticed ideological differences, there also exists the even more epidemiologically relevant group that is socially excluded and foregoes taking part in the democratic process. While it was possible to show some relevance of the anti-vaccine movement, the models imply that the key factor is not a recent lack of trust in COVID-19 vaccines as such, but decades-old lack of trust in the whole establishment. As a consequence, this means that the government should be very wary of well-intentioned pandemic policies that may be deemed overtly authoritarian and undermine the limited amount of trust even more. Instead, it should encourage those who are trusted by the least trustful parts of society to act.

## Figures and Tables

**Table 1:** Factors explaining vaccination rate in sub-regions for 30 June 2021.

Variable	Coefficient	Standard Error	*t*-Student	*p*-Value
Const	0.310	0.026	12.92	<0.001
Votes for Law and Justice	−0.201	0.017	−11.78	<0.001
Votes for Confederation	−1.678	0.168	−9.940	<0.001
2nd round voter turnout	0.308	0.050	6.130	<0.001
COVID-only deaths per 1000	0.015	0.004	3.663	<0.001
Employment-to-population ratio	0.133	0.053	2.207	0.013
Higher education	0.416	0.065	6.397	<0.001
Population density	1.004 × 10^−5^	3.872 × 10^−6^	2.593	0.010

**Table 2:** Factors explaining vaccination rate increase in sub-regions between 30 June and 31 August 2021 with prior vaccination level.

Variable	Coefficient	Standard Error	*t*-Student	*p*-Value
Const	0.114	0.007	15.41	<0.001
Vaccinated on 30 June 2021	0.060	0.012	5.089	<0.001
Votes for Confederation	−0.300	0.048	−6.148	<0.001
Votes for Kukiz	−0.120	0.016	−7.592	<0.001
Higher education	−0.107	0.017	−6.383	<0.001
People over 70	−0.173	0.031	−5.555	<0.001
People per vaccination site	9.349 × 10^−7^	3.329 × 10^−7^	2.809	0.005

**Table 3:** Factors explaining vaccination rate increase in sub-regions between 30 June and 31 August 2021 without prior vaccination level.

Variable	Coefficient	Standard Error	*t*-Student	*p*-Value
Const	0.141	0.005	25.88	<0.001
Votes for Confederation	−0.463	0.038	−12.12	<0.001
Votes for Kukiz	−0.105	0.016	−6.516	<0.001
Higher education	−0.047	0.012	−3.820	<0.001
People over 70	−0.206	0.032	−6.519	<0.001
People per vaccination site	1.377 × 10^−6^	3.319 × 10^−7^	4.148	<0.001

**Table 4:** Factors explaining vaccination rate in sub-regions for 30 June 2021 with spatial lag.

Variable	Coefficient	Standard Error	*t*-Student	*p*-Value
Vaccination rate in 6 nearest regions	0.528	0.039	13.53	<0.001
Const	0.132	0.026	5.112	<0.001
Votes for Law and Justice	−0.101	0.017	−6.143	<0.001
Votes for Confederation	−0.951	0.153	−6.188	<0.001
2nd round voter turnout	0.126	0.046	2.761	0.006
COVID-only deaths per 1000	0.019	0.004	5.300	<0.001
Employment-to-population ratio	0.031	0.046	0.674	0.500
Higher education	0.639	0.057	11.12	<0.001
Population density	3.040 × 10^−6^	3.347 × 10^−6^	0.908	0.363

**Table 5:** Factors explaining vaccination rate increase in sub-regions between 30 June and 31 August 2021 with prior vaccination level.

Variable	Coefficient	Standard Error	*t*-Student	*p*-Value
Vaccination rate increase in 6 nearest sub-regions	0.595	0.046	12.91	<0.001
Const	0.063	0.008	7.795	<0.001
Vaccinated on 30 June 2021	0.018	0.010	1.841	0.066
Votes for Confederation	−0.174	0.044	−3.929	<0.001
Votes for Kukiz	−0.057	0.014	−4.124	<0.001
Higher education	−0.070	0.013	−5.302	<0.001
People over 70	−0.114	0.027	−4.212	<0.001
People per vaccination site	3.042 × 10^−7^	2.831 × 10^−7^	1.075	0.282

## Data Availability

https://www.bdl.stat.gov.pl/BDL/dane/podgrup/temat; https://www.gov.pl/web/szczepimysie/mapa-punktow-szczepien; https://www.gov.pl/web/szczepienia-gmin/sprawdz-poziom-wyszczepienia-mieszkancow-gmin; https://www.gov.pl/web/szczepimysie/raport-szczepien-przeciwko-COVID-19; https://www.pkw.gov.pl.

## Abbreviations

COVID-19: the coronavirus disease 2019, European Union: EU, percentage points: p.p.

## References

[1] Raciborski F., Jankowski M., Gujski M., Pinkas J., Samel-Kowalik P. (2021). Changes in Attitudes towards the COVID-19 Vaccine and the Willingness to Get Vaccinated among Adults in Poland: Analysis of Serial, Cross-Sectional, Representative Surveys, January–April 2021. Vaccines.

[2] Sowa P., Kiszkiel Ł., Laskowski P.P., Alimowski M., Szczerbiński Ł., Paniczko M., Moniuszko-Malinowska A., Kamiński K. (2021). COVID-19 Vaccine Hesitancy in Poland-Multifactorial Impact Trajectories. Vaccines.

[3] Callaghan T., Moghtaderi A., Lueck J.A., Hotez P., Strych U., Dor A., Fowler E.F., Motta M. (2021). Correlates and Disparities of Intention to Vaccinate against COVID-19. Soc. Sci. Med..

[4] Reiter P.L., Pennell M.L., Katz M.L. (2020). Acceptability of a COVID-19 Vaccine among Adults in the United States: How Many People Would Get Vaccinated?. Vaccine.

[5] Fridman A., Gershon R., Gneezy A. (2021). COVID-19 and Vaccine Hesitancy: A Longitudinal Study. PLoS ONE.

[6] Malik A.A., McFadden S.M., Elharake J., Omer S.B. (2020). Determinants of COVID-19 Vaccine Acceptance in the US. EClinicalMedicine.

[7] Kreps S., Prasad S., Brownstein J.S., Hswen Y., Garibaldi B.T., Zhang B., Kriner D.L. (2020). Factors Associated with US Adults’ Likelihood of Accepting COVID-19 Vaccination. JAMA Netw. Open.

[8] Murphy J., Vallières F., Bentall R.P., Shevlin M., McBride O., Hartman T.K., McKay R., Bennett K., Mason L., Gibson-Miller J. (2021). Psychological Characteristics Associated with COVID-19 Vaccine Hesitancy and Resistance in Ireland and the United Kingdom. Nat. Commun..

[9] Hyland P., Vallières F., Shevlin M., Bentall R.P., McKay R., Hartman T.K., McBride O., Murphy J. (2021). Resistance to COVID-19 Vaccination Has Increased in Ireland and the United Kingdom during the Pandemic. Public Health.

[10] Edwards B., Biddle N., Gray M., Sollis K. (2021). COVID-19 Vaccine Hesitancy and Resistance: Correlates in a Nationally Representative Longitudinal Survey of the Australian Population. PLoS ONE.

[11] Afifi T.O., Salmon S., Taillieu T., Stewart-Tufescu A., Fortier J., Driedger S.M. (2021). Older Adolescents and Young Adults Willingness to Receive the COVID-19 Vaccine: Implications for Informing Public Health Strategies. Vaccine.

[12] Schwarzinger M., Watson V., Arwidson P., Alla F., Luchini S. (2021). COVID-19 Vaccine Hesitancy in a Representative Working-Age Population in France: A Survey Experiment Based on Vaccine Characteristics. Lancet Public Health.

[13] Domnich A., Cambiaggi M., Vasco A., Maraniello L., Ansaldi F., Baldo V., Bonanni P., Calabrò G.E., Costantino C., de Waure C. (2020). Attitudes and Beliefs on Influenza Vaccination during the COVID-19 Pandemic: Results from a Representative Italian Survey. Vaccines.

[14] La Vecchia C., Negri E., Alicandro G., Scarpino V. (2020). Attitudes towards Influenza Vaccine and a Potential COVID-19 Vaccine in Italy and Differences across Occupational Groups, September 2020. Med. Lav..

[15] Pivetti M., Melotti G., Bonomo M., Hakoköngäs E. (2021). Conspiracy Beliefs and Acceptance of COVID-Vaccine: An Exploratory Study in Italy. Soc. Sci..

[16] Soares P., Rocha J.V., Moniz M., Gama A., Laires P.A., Pedro A.R., Dias S., Leite A., Nunes C. (2021). Factors Associated with COVID-19 Vaccine Hesitancy. Vaccines.

[17] Hammer C.C., Cristea V., Dub T., Sivelä J. (2021). High but Slightly Declining COVID-19 Vaccine Acceptance and Reasons for Vaccine Acceptance, Finland April to December 2020. Epidemiol. Infect..

[18] Kourlaba G., Kourkouni E., Maistreli S., Tsopela C.-G., Molocha N.-M., Triantafyllou C., Koniordou M., Kopsidas I., Chorianopoulou E., Maroudi-Manta S. (2021). Willingness of Greek General Population to Get a COVID-19 Vaccine. Glob. Health Res. Policy.

[19] Lin Y., Hu Z., Zhao Q., Alias H., Danaee M., Wong L.P. (2020). Understanding COVID-19 Vaccine Demand and Hesitancy: A Nationwide Online Survey in China. PLoS Negl. Trop. Dis..

[20] Wang J., Jing R., Lai X., Zhang H., Lyu Y., Knoll M.D., Fang H. (2020). Acceptance of COVID-19 Vaccination during the COVID-19 Pandemic in China. Vaccines.

[21] Kadoya Y., Watanapongvanich S., Yuktadatta P., Putthinun P., Lartey S.T., Khan M.S.R. (2021). Willing or Hesitant? A Socioeconomic Study on the Potential Acceptance of COVID-19 Vaccine in Japan. Int. J. Environ. Res. Public Health.

[22] Neumann-Böhme S., Varghese N.E., Sabat I., Barros P.P., Brouwer W., van Exel J., Schreyögg J., Stargardt T. (2020). Once We Have It, Will We Use It? A European Survey on Willingness to Be Vaccinated against COVID-19. Eur. J. Health Econ..

[23] Lindholt M.F., Jørgensen F., Bor A., Petersen M.B. (2021). Public Acceptance of COVID-19 Vaccines: Cross-National Evidence on Levels and Individual-Level Predictors Using Observational Data. BMJ Open.

[24] Bono S.A., de Moura Villela E.F., Siau C.S., Chen W.S., Pengpid S., Hasan M.T., Sessou P., Ditekemena J.D., Amodan B.O., Hosseinipour M.C. (2021). Factors Affecting COVID-19 Vaccine Acceptance: An International Survey among Low- and Middle-Income Countries. Vaccines.

[25] Qunaibi E.A., Helmy M., Basheti I., Sultan I. (2021). A High Rate of COVID-19 Vaccine Hesitancy in a Large-Scale Survey on Arabs. eLife.

[26] Lazarus J.V., Wyka K., Rauh L., Rabin K., Ratzan S., Gostin L.O., Larson H.J., El-Mohandes A. (2020). Hesitant or Not? The Association of Age, Gender, and Education with Potential Acceptance of a COVID-19 Vaccine: A Country-Level Analysis. J. Health Commun..

[27] Trent M., Seale H., Chughtai A.A., Salmon D., MacIntyre C.R. (2021). Trust in Government, Intention to Vaccinate and COVID-19 Vaccine Hesitancy: A Comparative Survey of Five Large Cities in the United States, United Kingdom, and Australia. Vaccine.

[28] Pagliaro S., Sacchi S., Pacilli M.G., Brambilla M., Lionetti F., Bettache K., Bianchi M., Biella M., Bonnot V., Boza M. (2021). Trust Predicts COVID-19 Prescribed and Discretionary Behavioral Intentions in 23 Countries. PLoS ONE.

[29] Dodd R.H., Cvejic E., Bonner C., Pickles K., McCaffery K.J. (2021). Willingness to Vaccinate against COVID-19 in Australia. Lancet Infect. Dis..

[30] Hadjipanayis A., van Esso D., del Torso S., Dornbusch H.J., Michailidou K., Minicuci N., Pancheva R., Mujkic A., Geitmann K., Syridou G. (2020). Vaccine Confidence among Parents: Large Scale Study in Eighteen European Countries. Vaccine.

[31] Lazarus J.V., Ratzan S.C., Palayew A., Gostin L.O., Larson H.J., Rabin K., Kimball S., El-Mohandes A. (2021). A Global Survey of Potential Acceptance of a COVID-19 Vaccine. Nat. Med..

[32] De Figueiredo A., Simas C., Karafillakis E., Paterson P., Larson H.J. (2020). Mapping Global Trends in Vaccine Confidence and Investigating Barriers to Vaccine Uptake: A Large-Scale Retrospective Temporal Modelling Study. Lancet.

[33] Hornsey M.J., Harris E.A., Fielding K.S. (2018). The Psychological Roots of Anti-Vaccination Attitudes: A 24-Nation Investigation. Health Psychol..

[34] Sprawdź Poziom Wyszczepienia Mieszkańców w Gminach-Szczepienia Gmin-Portal Gov.pl. https://www.gov.pl/web/szczepienia-gmin/sprawdz-poziom-wyszczepienia-mieszkancow-gmin.

[35] GUS—Bank Danych Lokalnych. https://bdl.stat.gov.pl/BDL/dane/podgrup/temat.

[36] Basiw. https://basiw.mz.gov.pl/index.html#/visualization?id=3653.

[37] Mapa Punktów Szczepień—Szczepienie Przeciwko COVID-19—Portal Gov.pl. https://www.gov.pl/web/szczepimysie/mapa-punktow-szczepien.

[38] Raport Szczepień Przeciwko COVID-19—Szczepienie Przeciwko COVID-19—Portal Gov.pl. https://www.gov.pl/web/szczepimysie/raport-szczepien-przeciwko-COVID-19.

[39] Żuk P. (2020). Right-Wing Populism in Poland and Anti-Vaccine Myths on YouTube: Political and Cultural Threats to Public Health. Glob. Public Health.

[40] Wike R., Poushter J., Silver L., Cornibert S. (2019). Most Embrace Democracy and the EU, but Many Worry about the Political and Economic Future.

[41] Dane w Arkuszach—Wybory Prezydenta Rzeczypospolitej Polskiej w 2020 r. https://prezydent20200628.pkw.gov.pl/prezydent20200628/pl/dane_w_arkuszach.

[42] Korwin-Mikke: Mamy do Czynienia z Bandą Oszołomów, Która Chce Wszystkich Zaszczepić. https://www.gazetaprawna.pl/wiadomosci/artykuly/857638,korwin-mikke-mamy-do-czynienia-z-banda-oszolomow-ktora-chce-wszystkich-zaszczepic.html.

[43] Głosowanie Nr 75 Na 19. Posiedzeniu Sejmu. https://www.sejm.gov.pl/sejm9.nsf/agent.xsp?symbol=glosowania&nrkadencji=9&nrposiedzenia=19&nrglosowania=75.

[44] Bosak: Osoby, Które Uważają, Że to Jest Dla Nich Dobre Rozwiązanie, Pewnie Powinny Się Zaszczepić. https://polskieradio.pl/art299_2649570.

[45] Korwin-Mikke OSTRO! “Szczepienie dzieci to zbrodnia”. https://korwin24.pl/index.php/2021/07/30/korwin-mikke-ostro-szczepienie-dzieci-to-zbrodnia/.

[46] Kim Jest Wyborca Kukiza?. http://tajnikipolityki.pl/kim-jest-wyborca-kukiza/.

[47] Poseł Skutecki z Kukiz’15 na Marszu Antyszczepionkowców. Skarga do Komisji Etyki Poselskiej. https://www.medonet.pl/zdrowie,posel-skutecki-z-kukiz-15-na-marszu-antyszczepionkowcow--skarga-do-komisji-etyki-poselskiej,artykul,1725536.html.

[48] Feleszko W., Lewulis P., Czarnecki A., Waszkiewicz P. (2021). Flattening the Curve of COVID-19 Vaccine Rejection—An International Overview. Vaccines.

[49] Psychologiczne Charakterystyki Elektoratów, CBOS 2019. https://cbos.pl/SPISKOM.POL/2019/K_102_19.PDF.

[50] Brezzi M. (2020). All you need is trust: Informing the Role of Government in the COVID-19 Context. The OECD Statistics Newsletter.

[51] Rzymski P., Zeyland J., Poniedziałek B., Małecka I., Wysocki J. (2021). The Perception and Attitudes toward COVID-19 Vaccines: A Cross-Sectional Study in Poland. Vaccines.

[52] Cialdini R. (1993). Influence: Science and Practice.

[53] MacCoun R.J. (2012). The Burden of Social Proof: Shared Thresholds and Social Influence. Psychol. Rev..

[54] COVID-19 Vaccine Tracker | European Centre for Disease Prevention and Control. https://vaccinetracker.ecdc.europa.eu/public/extensions/COVID-19/vaccine-tracker.html#uptake-tab.

[55] Fact Check: Outdated Video of Fauci Saying “There’s No Reason to Be Walking around with a Mask”. https://www.reuters.com/article/uk-factcheck-fauci-outdated-video-masks-idUSKBN26T2TR.

[56] Szumowski o Koronawirusie: Maseczki nie Pomagają. 80 Proc. Zachorowań Przebiega w Sposób Łagodny. https://www.rmf24.pl/tylko-w-rmf24/poranna-rozmowa/news-szumowski-o-koronawirusie-maseczki-nie-pomagaja-80-proc-zach,nId,4347032.

[57] Gollust S.E., Nagler R.H., Fowler E.F. (2020). The Emergence of COVID-19 in the US: A Public Health and Political Communication Crisis. J. Health Polit. Policy Law.

[58] Noar S.M., Austin L. (2020). (Mis)Communicating about COVID-19: Insights from Health and Crisis Communication. Health Commun..

[59] Wang H., Cleary P.D., Little J., Auffray C. (2020). Communicating in a Public Health Crisis. Lancet Digit. Health.

[60] Xie Y., Peng S. (2009). How to Repair Customer Trust after Negative Publicity: The Roles of Competence, Integrity, Benevolence, and Forgiveness. Psychol. Mark..

[61] Claeys A.-S., Cauberghe V., Vyncke P. (2010). Restoring Reputations in Times of Crisis: An Experimental Study of the Situational Crisis Communication Theory and the Moderating Effects of Locus of Control. Public Relat. Rev..

[62] Lee S., Chung S. (2012). Corporate Apology and Crisis Communication: The Effect of Responsibility Admittance and Sympathetic Expression on Public’s Anger Relief. Public Relat. Rev..

[63] Zaufanie Społeczne. CBOS 2020. https://www.cbos.pl/SPISKOM.POL/2020/K_043_20.PDF.

